# Editorial: Osteoporosis secondary to endocrine disorders

**DOI:** 10.3389/fendo.2023.1194241

**Published:** 2023-05-24

**Authors:** Elżbieta Skowrońska-Jóźwiak, Krzysztof Lewandowski

**Affiliations:** Department of Endocrinology and Metabolic Diseases, Medical University of Lodz, Lodz, Poland

**Keywords:** secondary osteoporosis, diabetic bone disease, bone mineral density, fractures, bone metabolism

Though osteoporosis seems to belong to spectrum typically covered by orthopaedic surgeons, in fact bones represent a mirror that reflects a plethora of metabolic and endocrine processes. Hence, bone metabolism is altered not only in primary conditions, caused by aging and/or postmenopausal state but also in secondary conditions including endocrine disorders (1). In this issue we have therefore endeavoured to address the impact of multiple endocrine/metabolic abnormalities in relation to bone function. We hope that such approach would provide an opportunity to demonstrate a multidisciplinary nature of bone metabolic disorders in relation to multifaceted fields of clinical endocrinology.

Multiple endocrinopathies lead to osteoporosis development (1, 2). Some of them are common - like diabetes mellitus (DM), one of major chronic illness (3). In current issue this is addressed in a “*A narrative review of diabetic bone disease: Characteristics, pathogenesis, and treatment*” by Wu et al. The authors discuss the DM influence on bone mineral density, bone markers and fracture risk. Pathogenesis of diabetic bone disease and treatment of diabetic bone disease is also presented. The paper is of interests not only for endocrinologists, but also for other health specialists dealing with metabolic diseases.

Obesity is typically recognized as a factor known to be a protective factor against osteoporosis, though associated with an increased prevalence of osteoarthritis (4). However, recent studies have shown that excessive adiposity may be also detrimental for bone health. In the study by Charoenngam et al. entitled “*Increased Fat Mass Negatively Influences Femoral Neck Bone Mineral Density in Men but Not Women*” fat mass negatively correlated with bone mineral density of femoral neck, however this association was present only in men. Though this association did not seem to be particularly strong and so requires confirmation in further studies, it clearly challenges the established paradigm that “more adiposity means less osteoporosis”.

The growing prevalence of obesity leads to an increase in the incidence of metabolic dysfunction-associated fatty liver disease (MAFLD) (5, 6). Influence of MAFLD on bone mineral density was addressed by Li et al. in their paper on “*Association of metabolic dysfunction-associated fatty liver disease and liver stiffness with bone mineral density in American adults*”. The Authors concluded that MAFLD and liver stiffness were associated with higher femoral and lumbar bone mineral density in individuals aged over 50 years, but the results may be confounded by BMI. In our opinion this is an interesting observation given the high and ever increasing prevalence of MAFLD.

Excess of glucocorticosteroids is a classical adrenal reason of osteoporosis, however primary hyperaldosteronism can also affects bone metabolism (7, 8). Two manuscripts of this Research Topic tackle this issue, the first one by Lv et al. in their paper entitled “*Risk Factors Associated with Lower Bone Mineral Density in Primary Aldosteronism Patients*” and the second one in metaanalysis titled “*Bone and Mineral Metabolism in Patients with Primary Aldosteronism: A Systematic Review and Meta-Analysis*” by Wang et al. Authors show that aldosterone may affect bone metabolism by interacting with PTH or vitamin D. It remains to be established to what extent this interaction could be translated into clinically significant management problem.

X-linked congenital adrenal dysplasia is a rare inherited form of primary adrenal insufficiency, accompanying by hypogonadotropic hypogonadism (9). In the their paper entitled “*Clinical characteristics and treatment of secondary osteoporosis induced by X-linked congenital adrenal dysplasia*”, the Authors, namely Tao et al. share their experience in the treatment of this rare disease entity with bisphosphonates and vitamin K derivatives.

It is well known that thyroid dysfunction has detrimental effects on bone metabolism; especially overt hyperthyroidism increase bone turnover, leading to osteoporosis development (10, 11). However, the influence of TSH within the reference range on bone mineral density is not clear. In the study of Sheng et al. entitled “*T4 rather than TSH correlates with BMD among euthyroid adults*”, carried out in the group of euthyroid adults, T4 exhibited a negative correlation with BMD regardless of age and gender, both in subjects with normal and lowered BMD. Moreover, high normal FT4 was associated with an increased prevalence of previous fractures. TSH was not associated with variations of BMD and the fracture risk.

We hope that such broad range of conditions related to factors that influence the risk of osteoporosis would be of interests not only to physicians directly involved in the care of osteoporosis patients, but also to doctors and/or scientists who are usually involved the management of other endocrine and metabolic disorders.

## Author contributions

All authors listed have made a substantial, direct, and intellectual contribution to the work and approved it for publication.
